# Effects of a newborn care education program using ubiquitous learning on exclusive breastfeeding and maternal role confidence of first-time mothers in Vietnam: a quasi-experimental study

**DOI:** 10.4069/kjwhn.2021.12.03

**Published:** 2021-12-13

**Authors:** Tran Thi Nguyet, Nguyen Vu Quoc Huy, Yunmi Kim

**Affiliations:** 1 Department of Nursing, University of Medicine and Pharmacy, Hue University, Vietnam; 2 Department of Obstetrics and Gynecology, University of Medicine and Pharmacy, Hue University, Vietnam; 3 College of Nursing, Gachon University, Incheon, Korea

**Keywords:** Breast feeding, Infant health, Parturition, Smartphone, Vietnam

## Abstract

**Purpose:**

This study aimed to examine the effect of a newborn care education program using ubiquitous learning (UL-NCEP) on exclusive breastfeeding and maternal role confidence of first-time mothers in Vietnam.

**Methods:**

This quasi-experimental study with a nonequivalent control group design was conducted at a university hospital in Hue city, Vietnam, between June and July 2018. Eligible first-time mothers were conveniently allocated to the experimental (n=27) and the control group (n=25). Mothers in the control group received only routine care, whereas mothers in the experimental group received UL-NCEP through tablet personal computers in addition to routine care in the hospital. Then, the educational content was provided to mothers by their smartphone for reviewing at home. UL-NCEP was developed based on the World Health Organization’s “Essential Newborn Care Course” guidelines. The exclusive breastfeeding rate and maternal role confidence level after birth and at 4 weeks postpartum were assessed in both groups to assess the effect of UL-NCEP.

**Results:**

At 4 weeks postpartum, the experimental group showed a significantly higher level than the control, for exclusive breastfeeding rate (*p*<.05) as well as mean maternal role confidence (*p*<.05).

**Conclusion:**

UL-NCEP was a feasible and effective intervention in increasing first-time Vietnamese mothers’ exclusive breastfeeding rate and maternal role confidence level. This program may be integrated into routine care for postpartum mothers to promote mother and infant health among first-time mothers in Vietnam.

## Introduction

The neonatal period is one of the most critical life stages in which newborns face the highest risk of death in the first month of life. In 2018, the average global neonatal mortality rate was 18/1.000 live births, and the neonatal mortality rate in Vietnam was reported to be 11/1.000 live births [1].

The survival and health development of newborns depend on the quality of care provided by caregivers [2]. Mothers play an essential role in newborn care at home, and those who have a knowledge deficit of newborn care and inappropriate or potentially harmful traditional beliefs in childcare may adversely affect the health and development of infants [3,4]. Therefore, it is necessary to provide education programs related to newborn care for mothers to have appropriate knowledge and accurate practice skills for caring for babies. The education program should focus more on first-time mothers who lack knowledge of newborn care than on multiparous mothers [5].

The World Health Organization (WHO) developed guidelines for essential newborn care courses consisting of initiation and exclusive breastfeeding, umbilical cord care, eye care, thermal protection, immunization, and illness management [6]. This knowledge is necessary for women who are preparing to become mothers, especially first-time mothers. Mothers should know how to provide essential care for a newborn. Furthermore, their knowledge of signs of an illness can increase their confidence in caring newborns and reduce the use of an inappropriate traditional method in newborn care, thereby reducing the incidence of newborn illness and deaths [4,7].

Postpartum mothers face many difficulties in newborn care because of a lack of knowledge. Postpartum mothers at National Hospital in Kenya had gaps in knowledge of essential newborn care, including eye care, cord care, and vaccination [8]. A previous study of 339 Vietnamese postpartum mothers also showed that they had inadequate knowledge of newborn care: only 8.3% of mothers knew about umbilical cord care; 49.6% knew about two dangerous signs for newborns; 50% knew about neonatal jaundice; and 73.7% were worried about vaccination of their babies [9]. Besides, in recent years, the exclusive breastfeeding rate for infants younger than 6 months of age in Vietnam decreased by 24% in 2014 and 17% in 2018 [10]. Lack of a mother’s knowledge of newborn care can negatively influence the health and development of the infant. Providing newborn care education programs (NCEPs) for mothers by healthcare providers may fill these gaps in knowledge [11].

Previous studies have shown that NCEPs have a significantly positive impact on mothers and infants. There is evidence of enhanced infant and maternal health, increased maternal knowledge of newborn care and maternal confidence, reduced anxiety [11], improved exclusive breastfeeding duration [12], and increased maternal role confidence [13,14]. Various teaching methods have been applied to provide newborn care knowledge for mothers, including discussions and posters [11], telephone follow-up support [12], video-assisted teaching [15], and booklets [14]. However, these methods may be limited to access by time, space. Therefore, it cannot always satisfy the various learning needs of mothers. However, the ubiquitous learning method can meet those requirements. In developing countries with unstable power supplies, ubiquitous learning with tablets is a new learning method that enables anyone to access at any time and anywhere through smart devices without limitations of time, place, or environment [16].

In Vietnam, newborns usually receive care from midwives during a hospital stay under the rooming-in system. After being discharged, care is mainly provided by the mother and their family members at home. However, almost all mothers do not receive any education programs for newborn care at home. In Vietnamese culture, mothers should be at home during the first month postpartum, so it is important to provide an ubiquitous learning method (UL-NCEP) for mothers to help them care for their newborn at home. Research to develop and evaluate the effectiveness of the NCEP is still limited in Vietnam, and almost no studies have used the ubiquitous learning method for health education for newborn care.

This study aimed to examine the effect of a UL-NCEP on the exclusive breastfeeding rate and maternal role confidence level of first-time Vietnamese mothers. It was hypothesized that (1) the exclusive breastfeeding rate and (2) the maternal role confidence level would be significantly higher in the experimental group participating in the UL-NCEP than in the control group.

## Methods

Ethics statement: This study was approved by the Institutional Review Board of Hue University of Medicine and Pharmacy (No. H2018/351). Informed consent was obtained from the participants.

### Study design

This quasi-experimental study with a nonequivalent control group design was conducted to examine the effect of a UL-NCEP on the exclusive breastfeeding rate and maternal role confidence level of first-time mothers. This study report followed the TREND (Transparent Reporting of Evaluations with Non-randomized Designs) reporting guidelines [17].

### Setting and participants

This study included first-time mothers from the Obstetrics and Gynecology Department of Hue University of Medicine and Pharmacy’s Hospital in Vietnam between June and July 2018. The inclusion criteria of the study were as follows: (1) first-time Vietnamese mothers who were inpatient for 3 days after vaginal birth or 5 days after cesarean section; (2) at least 18 years of age; (3) who delivered a healthy newborn (full-term, Apgar score of ≥7, and no birth defects); (4) had no history of obstetric, medical, and psychological problems; (5) did not receive an education program related to newborn care; and (6) understood the study’s purpose and agreed to participate in this study. The exclusion criteria were as follows; (1) mothers and newborn who had any postpartum health problems and (2) those who did not agree to participate.

### Study size

**Figure 1.**
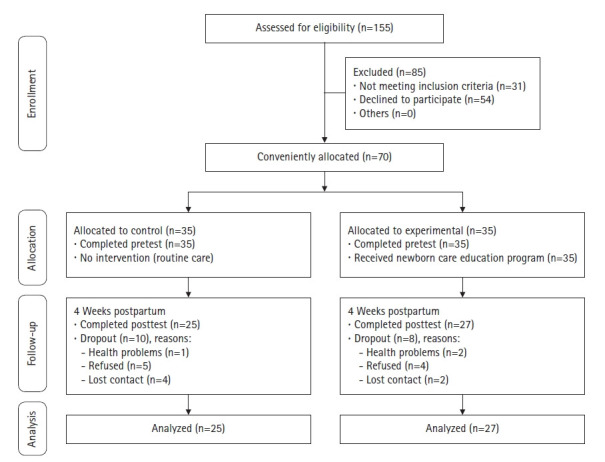
Flow diagram of the study.

The sample size of this study was calculated using the G*Power 3.0.10 program. With a power of 0.80, an alpha set at 0.05, and an effect size of 0.5, the minimum sample size required 52 participants. Considering that the dropout rate in Vietnam may be higher than in Korea [13], 35 mothers were included in each group. This research started with 70 mothers, but a total of 52 mothers completed the study (27 in the experimental group and 25 in the control group). A convenience sampling method was used to assign mothers to the experimental and control groups. The participant flow diagram is shown in Figure 1.

### Measurements/instruments

The following instruments were used in this study; general characteristics, exclusive breastfeeding rate, and maternal role confidence level questionnaires.

#### Exclusive breastfeeding rate

We asked mothers how they were feeding their babies and the reason for non-exclusive breastfeeding. Feeding type included exclusive breastfeeding, mixed feeding, and exclusive formula. In this study, exclusive breastfeeding refers to feeding breast milk without other food or drinks, even water.

#### Maternal role confidence scale

The maternal role confidence scale was modified and supplemented by Jang [18], and it was based on 14 items in Parker and Zahr’s “Maternal Confidence Questionnaire” [19]. Then, the scale was translated into Vietnamese in Lee and Oh’s study [14]. The composition of the scale includes six fields: comfort and safety, infant behavior, lactation, hygiene, bathing and changing a diaper, and health and illness; the maternal role confidence scale consists of 35 items, each of which is rated on a 5-point Likert scale ('almost no confidence' 1, to 'very confident' 5). The total score ranges from 35 to 175, with higher scores indicating a higher level of maternal role confidence. The Cronbach’s α values of the scale were in .85 [19], .92 [14], and .95 in the present study.

#### General characteristics

This questionnaire consisted of eight questions for determining socio-demographic characteristics such as age, educational level, occupation, family status, average income, region of residence, and obstetric characteristics, such as the delivery method and sex of the baby.

### Data collection/procedure

All mothers in the study were informed of the purpose of the research before data collection. To prevent the spread of UL-NCEP, data collection on the control group was performed first. Data were collected using the aforementioned questionnaires about general characteristics, breastfeeding, and maternal role confidence. The pretest data were collected in the hospital within 24 to 72 hours after the mothers gave birth.

After the pretest was administered, the control group received routine care during hospitalization from midwives in the Obstetrics and Gynecology Department. Routine care included newborn care such as baby bathing, umbilical cord care, hepatitis B vaccination for high-risk babies, and maternal care, such as incision care, medication, and neonatal screening consultants. The routine care did not provide systematic knowledge of newborn care at home to mothers. However, about 20% of mothers from this hospital choose paid home care service. The mothers in this study consisted of those who did not choose home care services.

The experimental group received the NCEP in addition to routine care. The NCEP was developed based on the WHO “Essential Newborn Care Course” guidelines [6], a reference manual for “Care of the Newborn” [20], an extensive review of the literature, and a newborn care program developed in a previous study [13,14]. The newborn care education content consisted of five topics for essential newborn care as follows: (1) breastfeeding, (2) sleep and activities, (3) environment and safety, (4) hygiene, and (5) managing newborn health. The NCEP is presented in detail in Table 1. The contents of the program were evaluated by two experts in infant care and were modified according to their advice. The content of the NCEP was designed using text, 18 pictures, and four simulation videos that helped mothers understand the material easily. Mothers were encouraged to self-study how to care appropriately for their babies. A ubiquitous learning method using tablet personal computers (PCs) was used to provide an NCEP to mothers. After they completed the pretest, the NCEP was provided face-to-face from the mother’s bed through tablets PCs provided by research team. The NCEP was implemented when mothers felt comfortable and before they were discharged. While educating, questions and answers were encouraged to increase the interaction between one researcher (author A) and mother. Before discharge, PDF materials of NCEP were downloaded to them by their smartphone so that they could use NCEP even when they went home. A researcher checked mothers whether the newborn was cared for by studying the smartphone data every week through she got the mothers’ text messages.

At 4 weeks postpartum, the posttest data were collected in the homes of mothers for both groups using the same instruments for the pretest by the researcher visiting their home.

### Data analysis

Descriptive statistics, the independent t-test, chi-square test, and Fisher’s exact test were used to identify the homogeneity of participants’ characteristics and compare outcome baseline variables between the two groups. The chi-square test was used to evaluate the difference of the exclusive breastfeeding rate at posttest between the two groups to assess the effect of the NCEP on maternal role confidence, the paired sample t-test was used to compare the difference between pretest and posttest responses in each group, and an independent sample t-test was used to compare the difference of pretest and posttest responses between the experimental and control groups. A *p*-value of <.05 was considered statistically significant, and the confidence interval was 95%. The reliability of the research instruments was verified using Cronbach’s α values. All collected data were analyzed using IBM SPSS for Windows, ver. 20.0 (IBM Corp., Armonk, NY, USA).

## Results

### Comparison of general characteristics and outcome variables

The general characteristics of mothers in the experimental (n=27) and control (n=25) groups are shown in Table 2.

In the experimental group, 92.6% of mothers were younger than 30 years of age, 48.1% had more than college level education, 66.7% had full-time jobs, 70.4% were living with extended family, 59.3% were living in urban areas, 55.6% had cesarean section, and 66.7% had male babies. In the control group, 96.0% of mothers were younger than 30 years of age, 40.0% had more than college level education, 60.0% had full-time jobs, 72.0% were living with extended family, 60.0% were living in urban areas, 40.0% had cesarean section, and 60.0% had male babies. However, there were no significant differences in age, education level, occupation, family status, average income, region of residence, delivery method, and sex of the baby between the two groups.

At the pretest, the outcome variables showed no significant differences between the groups. As Table 2 shows, the exclusive breastfeeding rates of the experimental and control groups were only 14.8% and 12.0%, respectively. Maternal role confidences were at midpoint levels in both groups, 106.00±24.07 in the experimental and 103.06±20.64 in the control group.

### Comparison of the exclusive breastfeeding rate

The exclusive breastfeeding rate of the experimental group at 4 weeks postpartum was 74.1%, which was higher than that of the control group (44.0%). The difference between the two groups was statistically significant (*χ*^2^=4.88, *p*=.027) (Table 3).

The reasons for stopping exclusive breastfeeding in both groups are listed in Table 4. Mother’s perception of insufficient milk (85.7%), baby’s refusal (9.5%), and maternal sickness (the common cold) (4.8%) were the main reasons given. In the experimental group, the reason for stopping exclusive breastfeeding was mother’s perception of insufficient milk (33.3%). However, in the control group, the reasons for stopping exclusive breastfeeding were mother’s perception of insufficient milk (52.3%), baby’s refusal of the breast (9.5%), and maternal sickness (the common cold) (4.8%).

### Comparison of the maternal role confidence level

Comparing posttest differences in the mean maternal role confidence level between the two groups, the experimental group (138.37±18.26) was statistically higher than that in the control group (111.60±22.74), and the difference between the two groups was statistically significant (t=–4.70, *p*<.001) (Table 5).

## Discussion

The NCEP about essential newborn care is a way for mothers to improve their cognitive knowledge and develop skills in newborn care. This is the first experimental study to develop an NCEP for promoting the health of Vietnamese mothers and babies.

This study’s results showed that the UL-NCEP has a significantly positive effect on exclusive breastfeeding at 4 weeks postpartum among first-time Vietnamese mothers. The NCEP in this study emphasized the benefits of exclusive breastfeeding for both the health of infants and mothers. Therefore, mothers perceived the importance of exclusive breastfeeding and changed their breastfeeding intention, finally increasing exclusive breastfeeding practices [21]. Telephone-based interventions (including text messages) have been a cost-effective way to provide community-based care, particularly to mothers in rural and remote areas [12]. In this study, education was provided more effectively by using smartphones, and it can be said that it is a cost-effective method similarly in the area of providing community-based care.

Moreover, the UL-NCEP had a significant effect on the maternal role confidence level at 4 weeks postpartum among first-time mothers. These results indicated that during the postpartum period, the UL-NCEP increased the maternal role confidence level of first-time mothers. This finding supports other those of previous studies that found that an NCEP before discharge increased maternal role confidence among primiparous women from Nepal [11], and a newborn parenting program significantly improved maternal role confidence of married immigrant primiparas women including Vietnamese women in Korea [14]. Providing an NCEP has been found to increase the confidence of mothers and may facilitate caring for their newborns and their adaptation to the role of mothers [22]. Moreover, face-to-face education during hospitalization and continued receipt of the educational program by using the ubiquitous learning method that was supportive helped the mothers in this study have confidence in providing newborn care in the postpartum period. With the ubiquitous learning method, although mothers received the same educational program whenever they needed even when they returned home, and it may have helped them increase their material role confidence. In this sample of Vietnamese mothers, ubiquitous learning was also feasible and applicable.

This study has some limitations. First, randomization was not possible. The control group was first investigated because of the prevention of the spread of the educational program. Second, mothers of this hospital were not representative of all new Vietnamese mothers. Third, we only performed the posttest once at 4 weeks postpartum; thus, the long-term effect of the UL-NCEP on exclusive breastfeeding and maternal role confidence of first-time mothers could not be evaluated. In the future, research is needed to confirm the long-term effect of UL-NECP.

This study showed that a UL-NCEP improved the exclusive breastfeeding rate and may have helped with the maternal role confidence in newborn care of first-time Vietnamese mothers. Therefore, for Vietnamese mothers, ubiquitous learning is feasible and applicable. The findings of the study are meaningful in that nursing and midwives could integrate this education program into routine care for postpartum mothers to promote mother and infant health among first-time mothers.

## Figures and Tables

**Table 1.** The ubiquitous learning-based newborn care education program contents

Topic	Contents	Time (minute)
1. Breastfeeding	Breastfeeding benefits	30
	Breastfeeding timeline	
	Latching and breastfeeding position	
	Breastfeeding simulation video	
2. Sleep and activities	Sleeping care	15
	Crying baby: reasons and handling	
	Holding and talking to the baby	
3. Environment and safety	Room temperature and humidity	15
	Maintaining warm/Kangaroo care	
4. Hygiene	Baby bathing and simulation video	15
	Umbilicus care and simulation video	
	General hygiene	
5. Managing newborn health	Dangerous sign in newborn	15
	Newborn jaundice	
	Hiccup and vomiting handling	
	Temperature measure and simulation video, fever management	
	Diarrhea sign and prevention	
	Vaccination	
Total		90

**Table 2.** Homogeneity test of general characteristics and outcome variables between two groups

Characteristic	n (%) or mean±SD		*χ*^2^/t/U	*p*
	Experimental (n=27)	Control (n=25)		
Age (year)				
<30	25 (92.6)	24 (96.0)	1.00^†^	.529
≥30	2 (7.4)	1 (4.0)		
Education level				
Elementary school	3 (11.1)	0 (0)	4.52^†^	.194
Secondary school	5 (18.5)	4 (16.0)		
High school	6 (22.2)	11 (44.0)		
≥College	13 (48.1)	10 (40.0)		
Occupation				
Full-time	18 (66.7)	15 (60.0)	0.40^†^	.942
Part-time	4 (14.8)	5 (20.0)		
None	5 (18.5)	5 (20.0)		
Family state				
Nuclear family	8 (29.6)	7 (28.0)	0.02	.897
Extended family	19 (70.4)	18 (72.0)		
Average monthly income (×million VND)	4.47±1.94	4.97±2.01	–.75^‡^	.453
Region				
Rural	11 (40.7)	10 (40.0)	0.00	.957
Urban	16 (59.3)	15 (60.0)		
Delivery method				
Vaginal delivery	12 (44.4)	15 (60.0)	1.26	.262
Cesarean section	15 (55.6)	10 (40.0)		
Baby sex				
Male	18 (66.7)	15 (60.0)	.25	.618
Female	9 (33.3)	10 (40.0)		
Breastfeeding practice				
Exclusive breastfeeding	4 (14.8)	3 (12.0)	1.68^†^	.477
Mixed feeding	15 (55.6)	18 (72.0)		
Formula feeding	8 (29.6)	4 (16.0)		
Maternal role confidence	106.00±24.07	103.04±20.64	–0.47	.637

VND: Vietnamese dollars (1 million VND is approximately 44 US dollars).

† Fisher’s exact test,

‡ Mann-Whitney U test

**Table 3.** Comparison of breastfeeding practice at 4 weeks postpartum

Feeding type	n (%)		*χ* ^2^	*p*
	Experimental (n=27)	Control (n=25)		
Exclusive breastfeeding	20 (74.1)	11 (44.0)	4.88	.027
Mixed feeding	7 (25.9)	14 (56.0)		

**Table 4.** Reason for stopping exclusive breastfeeding at 4 weeks postpartum

Reason	n (%)		
	Total (N=21)	Experimental (n=7)	Control (n=14)
Perceived insufficient milk	18 (85.7)	7 (33.3)	11 (52.3)
Baby’s refusal	2 (9.5)	0 (0)	2 (9.5)
Mother’s sickness (common cold)	1 (4.8)	0 (0)	1 (4.8)

**Table 5.** Comparison of maternal role confidence at 4 weeks postpartum

Group	Mean±SD		Difference, mean (95% Cl)	t	*p*
	Pretest	Posttest			
Control (n=25)	103.04±20.64	111.60±22.74	–8.56 (–15.27 to –2.85)	–2.63	.015
Experimental (n=27)	106.00±24.07	138.37±18.26	–32.37 (–41.76 to –22.98)	–7.09	<.001

CI: Confidence interval.
